# p16^INK4A^ Positively Regulates Cyclin D1 and E2F1 through Negative Control of AUF1

**DOI:** 10.1371/journal.pone.0021111

**Published:** 2011-07-20

**Authors:** Huda H. Al-Khalaf, Dilek Colak, Maher Al-Saif, Albandary Al-Bakheet, Siti-Faujiah Hendrayani, Nujoud Al-Yousef, Namik Kaya, Khalid S. Khabar, Abdelilah Aboussekhra

**Affiliations:** 1 Department of Biological and Medical Research, King Faisal Specialist Hospital and Research Center, Riyadh, Saudi Arabia; 2 Department of Biostatistics, Epidemiology and Scientific Computing, King Faisal Specialist Hospital and Research Center, Riyadh, Saudi Arabia; 3 Program in Biomolecular Research, King Faisal Specialist Hospital and Research Center, Riyadh, Saudi Arabia; 4 Department of Genetics, King Faisal Specialist Hospital and Research Center, Riyadh, Saudi Arabia; Cleveland Clinic, United States of America

## Abstract

**Background:**

The cyclin-D/CDK4,6/p16^INK4a^/pRB/E2F pathway, a key regulator of the critical G1 to S phase transition of the cell cycle, is universally disrupted in human cancer. However, the precise function of the different members of this pathway and their functional interplay are still not well defined.

**Methodology/Principal Findings:**

We have shown here that the tumor suppressor p16^INK4a^ protein positively controls the expression of cyclin D1 and E2F1 in both human and mouse cells. p16^INK4a^ stabilizes the mRNAs of the corresponding genes through negative regulation of the mRNA decay-promoting AUF1 protein. Immunoprecipitation of AUF1-associated RNAs followed by RT-PCR indicated that endogenous AUF1 binds to the cyclin D1 and E2F1 mRNAs. Furthermore, *AUF1* down-regulation increased the expression levels of these genes, while concurrent silencing of *AUF1* and p16^INK4a^, using specific siRNAs, restored normal expression of both cyclinD1 and E2F1. Besides, we have shown the presence of functional AU-rich elements in the E2F1 3′UTR, which contributed to p16/AUF1-mediated regulation of E2F1 post-transcriptional events *in vivo*. Importantly, genome-wide gene expression microarray analysis revealed the presence of a large number of genes differentially expressed in a p16^INK4a^ -dependent manner, and several of these genes are also members of the AUF1 and E2F1 regulons. We also present evidence that E2F1 mediates p16-dependent regulation of several pro- and anti-apoptotic proteins, and the consequent induction of spontaneous as well as doxorubicin-induced apoptosis.

**Conclusion/Significance:**

These findings show that the cyclin-dependent kinase inhibitor p16^ INK4a^ is also a modulator of transcription and apoptosis through controlling the expression of two major transcription regulators, AUF1 and E2F1.

## Introduction

The cyclin D-CDK4,6/p16/pRB/E2F cascade has been found to be altered in more than 80% of human tumors [1], [2]. Indeed, the disruption of this pathway is a mandatory requirement for 100% of lung cancer [3]. During G1 phase, pRB is inactivated by sequential phosphorylation events mediated by various cyclin-dependant kinases (CDKs) leading to the release of the E2F transcription factors, the activation of many genes and progression of the cell cycle [4]. Indeed, activation of the E2F1 transcription factor leads to coordinated induction of multiple downstream effectors implicated in both cell proliferation and death [5], [6]. Interestingly, E2F1 may function as an oncogene or as a tumor suppressor gene, and it seems that the threshold level of the E2F1 protein as well as the cell type have a determinant role in the function of the gene [5].

p16^INK4A^ (hereafter referred to as p16) is a cyclin-dependent kinase inhibitor that plays important roles in tumor suppression [7], [8], [9], [10]. The p16 coding-gene has been found homozygously deleted, mutated or transcriptionally inhibited by methylation in a large number of different human tumor types [8], [9], [11], [12]. Mice lacking p16 are tumor prone and develop different types of cancer, particularly after exposure to carcinogens [13], [14]. Furthermore, p16 promoter methylation and transcriptional silencing have been detected even in histologically normal mammary tissue of cancer-free women. This suggests that p16 inactivation may represent a cancerous precondition and an early event in promoting genomic instability that leads to tumorigenesis [15].

Recent data have revealed the existence of interactions between the various genes of the pRB pathway. Indeed, it has been reported that the expression of 74 genes is repressed by both p16 and pRB [16]. It has also been shown that the cyclin D1 protein stimulates E2F1 [17], [18]. Furthermore, over-expression of cyclin D1 contributed to malignancy by up-regulation of FGFR1 via the pRB/E2F pathway [19]. In the present report we addressed the role of p16 in the regulation of cyclin D1 and E2F1, two major players in the pRB pathway. We have shown that the transcription factor E2F1 is also a target of the RNA binding AUF1 protein, and that the tumor suppressor p16 positively controls cyclin D1 and E2F1 through negative regulation of AUF1. Furthermore, we provide clear evidence that p16 is also a master regulator of gene expression and apoptosis in human cells.

## Materials and Methods

### Cell lines, cell culture and chemicals

U2OS, EH1 and EH2 [20] (The three cell lines are a generous gift from Dr. G. Peters), MEFs p16 (WT) and their p16-specific knockout counterpart [13], Huh7 (hepatocarcinoma cell line) and HFSN1 (primary normal human skin fibroblast) [21]. These cells were routinely cultured in DMEM medium supplemented with 10% FBS. Actinomycin D, IPTG, Flavopiridol and doxorubicin were purchased from Sigma (USA).

### Cellular lysate preparation and immunobloting

This has been performed as previously described [20]. Antibodies directed against p16 (50.1), Cyclin D1 (HD11), E2F1 (KD95), hnRNP DO (T10), HuR (3A2), α-tubulin (TU-02), PCNA (PC-10), Bax (B-9), Bcl-2 (C-2), NF-κB (F-6), Bcl-xL (H-5), GAPDH (FL-335), Cdk4 (C-22), Cdk6 (H-11) and β-actin (C-11) were purchased from Santa Cruz (USA) and Cleaved Caspase-3 (Asp-175) from Cell signaling (UK).

### RNA purification, RT-PCR and real time RT-PCR

Total RNA was purified using the TRI reagent (Sigma) according to the manufacturer's instructions. The concentration of RNA was determined using NanoDrop® ND-1000 Spectrophotometer (Nanodrop Inc., Wilmington, DE, USA). Single stranded complementary DNA (cDNA) was obtained from reverse transcription of 1 µg of RNA using the RT-PCR kit (BD Biosciences) and following the manufacturer protocol. cDNA was then amplified with 1U Taq polymerase, dNTPs (50 mM), and primers (25 pmol each). The mixture was first heated at 94°C for 5 min and then 30 cycles at 94°C for 1 min, 55°C for 1 min and 72°C for 1 min, then 72°C for 10 min. PCR products were seen after electrophoresis on ethidium bromide stained 2% agarose gel. For real time RT-PCR Syber green and platinum Taq polymerase (Invitrogen) were used and the amplifications were performed utilizing the Bio-Rad iQ5 multicolor Real time PCR detection system.

The respective primers were:

β-actin: 5′-CCCAGCACAATGAAGATCAAGATCAT-3′ (forward) and 5′-ATCTGCTGGAAGGTGGACAGCGA-3′ (reverse); *cyclinD1*: 5′-CACACGGACTACAGGGGAGT-3′ (forward) and 5′-CACAGGAGCTGGTGTTCCAT-3′ (reverse); *E2F1*: 5′-ATGTTTTCCTGTGCCCTGAG-3′ (forward) and 5′-ATCTGTGGTGAGGGATGAGG-3′ (reverse); GAPDH: 5′-GAGTCCACTGGCGTCTTC-3′ (forward) and 5′-GGGGTGCTAAGCAGTTGGT-3′ (reverse); *AUF1:* 5′-GATCAAGGGGTTTTGGCTTT-3′ (forward) and 5′-GTTGTCCATGGGGACCTCTA-3′ (reverse); *p16*: 5′-CAACGCACCGAATAGTTACG-3′ (forward) and 5′-CAGCTCCTCAGCCAGGTC-3′ (reverse).

The intensity of the bands was determined with the Quantity One program (Bio-RAD) and was normalized against β-actin or GAPDH.

### Analysis of mRNA stability

Cells were challenged with 5 µg/ml Actinomycin D for various periods of time (0-6 hrs) and then total RNA was purified and assessed using real time RT-PCR.

### Sub-cellular fractionation

Nuclear and cytoplasmic extracts were prepared as previously described [21]. Briefly, cytoplasmic fractions were obtained by incubating cells in 200 µl of hypotonic buffer A (10 mM HEPES [pH 7.9], 10 mM KCl, 1.5 mM MgCl2) supplemented with protease inhibitors cocktail on ice, and lysed by addition of 25 µl of buffer A containing 2.5% Nonidet P-40 plus inhibitors. Nuclei were pelleted (3500 rpm, 4 min, 4°C), and supernatants were saved, freezed-thawed five times, and centrifuged (10 min, 3,500 rpm, 4°C). Nuclear pellets were incubated in extraction buffer C (20 mM HEPES [pH 7.9], 0.45 M NaCl, 1 m MEDTA) plus inhibitors and centrifuged (10 min, 14000 rpm, 4°C) and supernatants were saved at −80°C.

### Immunoprecipitation and RT-PCR

Cell lysates were prepared from confluent cells, and 3 mg were incubated in the lysis buffer (50 mM Tris (pH 8), 100 mM NaCl, 10% glycerol, 1× protease inhibitors, 5 mM DTT and 2 U/µl RNasin) and 5 µg AUF1 mouse monoclonal antibody (mouse IgG1 was used as control) was added and mixed at 4°C for 4 h. Equal volume of protein A agarose was added per immunoprecipitation and mixed overnight at 4°C. After centrifugation, the pellet was re-suspended in 1 ml TRI reagent used for RNA extraction. RT–PCR reactions were performed as described above.

### siRNA transfection

pSILENCER-*AUF1*siRNA and control-siRNA plasmids were used to carry out transient transfection using human dermal fibroblast nucleofector kit (Amaxa Biosystems) following the protocol recommended by the manufacturer.

pIRES.E2F1 and the scrambled-sequence plasmids were used to perform stable transfection of U2OS cells. Transfection was carried out by mixing 3 µg of the plasmid DNA in 1.5 ml of Opti-MEM I medium without serum. A mixture of 1.5 ml of Opti-MEM I medium with 36 µl Lipofectamine (invitrogen) was added to the DNA, and then was incubated for 20 min before addition to 50% confluent cells.

### 3′ UTR PCR, construction and cloning of reporter plasmids

The ARE and ARE-like sequences in the E2F1 3′UTR (1455–2700 nt, NM_005225.2) were identified by searching for AUUUA patterns. Double-stranded DNA was made by annealing oligonucleotides (80 nt) that correspond to sense and antisense orientation of the ARE regions were synthesized (Metabion, Germany) and contain BamHI and XbaI overhangs, respectively. The DNA was cloned into EGFP expression vector (Genelantis, San Diego, CA), which is under CMV constitutive promoter, and verified by specific PCR and sequencing.

### Reporter transfection and activity assessment

Reporter constructs containing GFP-3′UTR/ARE were used in transient transfection using Lipofectamine 2000 (Invitrogen). Transfection efficiency and normalization to control was achieved using GFP reporter fused with stable non-ARE BGH 3′UTR. The intra-well variance of any replicate groups in fluorescence is generally <6%, which does not warrant intra-well normalization of transfection [22]. Data are presented as mean value ± SEM of fluorescence intensity using BD Bioimager and imaging software.

### Apoptosis analysis by Annexin V/Flow Cytometry

Cells were either not treated or challenged with Doxorubicin. Detached and adherent cells were then harvested after 72 hrs, centrifuged and resuspended in 1 ml phosphate buffered saline (PBS). Cells were then stained with propidiom iodide (PI) and Alexa Flour 488 annexin V and analyzed as previously described [23].

### Quantification of protein expression level

The expression levels of the immunoblotted proteins were measured using the densitometer (BIO-RAD GS-800 Calibrated Densitometer) as previously described [20].

### Affymetrix Genechip Array Experiments

Genome-wide gene expression studies was performed using Affymetrix's latest GeneChip® Human Genome U133 Plus 2.0 Array which have more than 54,000 probe sets used to analyze the expression level of more than 47,000 transcripts and variants, including approximately 38,500 well-characterized human genes. To this end, double-stranded cDNA were made from total RNA using the Superscript cDNAs synthesis kit as recommended by the manufacturer (Invitrogen, Carlsbad, CA, USA). The product of this reaction was used as a template for in vitro transcription to make biotin-labeled cRNA (target cRNA) using biotinylated UTP and CTP. Target cRNA was fractionated and then hybridized to the gene chips for 16 hrs. The experimental procedures and quality control procedures at each critical step (before hybridization as well as post-hybridization) were strictly followed according to manufacturer's instructions. Washing, staining, and scanning were performed using Affymetrix's Fluidics Station 450 and GCS 3000 G7, respectively, according to the manufacturer's instructions and guidelines. All the necessary steps related to Affymetrix Fluidics Station 450 and GCS 3000 G7 were controlled by Affymetrix's GeneChip Operating Software (GCOS).

### Validation of the transcriptomic data by Real time RT-PCR (qRT-PCR)

To validate the microarray results, the expression levels of differentially expressed transcripts of randomly chosen 11 genes were determined by qRT-PCR experiments using ABI 7500 Sequence Detection System (Applied Biosystems, Foster City, CA, USA). To this end, 50 ng of total RNA procured from the same microarray study samples were transcribed into cDNA using cDNA Archive Kit (Applied Biosystems), and then used as template for PCR reaction using specific primers for the candidate genes, and GAPDH was used as internal control. Quantitech SYBR green (Qiagen) was used for detection and analysis. All reactions were conducted in triplicates and the data was analyzed using the delta delta CT method (Livak KJ, Schmittgen TD).

### Microarray analysis

The dChip outlier detection algorithm was used to identify outlier arrays [24]. All samples/chips passed the quality controls. The open source R/Bioconductor packages were used for processing and analysis of microarray data [25], which was normalized by the GC Robust Multi-array Average (GC-RMA) algorithm [26], [27]. Significantly modulated genes were defined as those with absolute fold change (FC) ≥2.0 and adjusted *p*-value<0.05. Statistical analyses were performed with the MATLAB software packages (Mathworks, Natick, MA, USA), and PARTEK Genomics Suite (Partek Inc., St. Lois, MO, USA).

## Results

### p16 modulates the expression of cyclin D1 and E2F1 in both human and mouse cells

**Figure 1:**
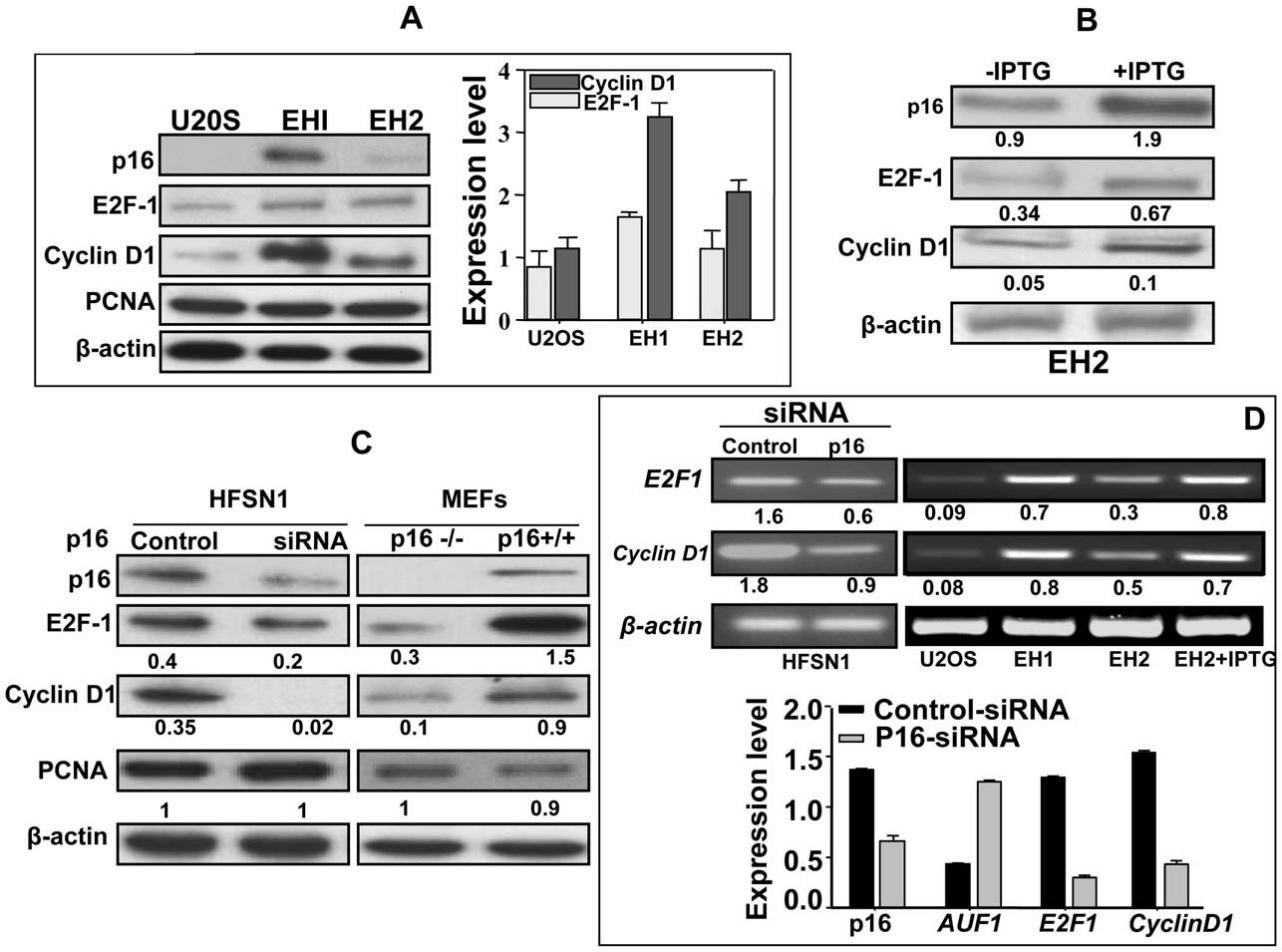
p16 modulates E2F1 and cyclin D1 protein and mRNA levels in human and mouse cells. Whole cell extracts and total RNA were prepared from different human and mouse cell lines. (A–C) Western blots using the indicated antibodies. The histogram shows the expression levels of the indicated proteins. EH2 cells were treated with IPTG at 1 mM (D) Upper panel, ethidium bromide stained agarose gels showing RT-PCR products of the indicated genes. The numbers below the bands indicate the corresponding expression levels relative to β-actin. These experiments were repeated several times and representative ones are shown. The histogram shows data of real time RT-PCR of the indicated genes. Error bars indicate standard errors of 3 different experiments.

We analyzed the effect of p16 on the expression levels of E2F1 and cyclin D1 in both human and mouse cells. First, we used the p16-defective osteosarcoma U2OS cell line and its derivatives EH1 and EH2 that express p16 under the control of an IPTG inducible promoter [28]. The p16 expression in these cells does not exert any measurable effect on cellular growth [28]. Whole cell extracts from U2OS, EH1 and EH2 cells were prepared and used to analyze the levels of the E2F1 and cyclin D1 proteins using specific antibodies. Figure 1A shows that the protein levels of E2F1 and cyclin D1 are modulated in a p16-dependent manner. Interestingly, IPTG-treated EH2 cells wherein p16 protein level was augmented, the expression levels of the cyclin D1 and E2F1 proteins increased 2 fold as compared to the non-treated cells (Figure 1B). To further elucidate the p16 effect on the expression of these proteins, we evaluated the levels of cyclin D1 and E2F1 in HFSN1 cells stably expressing either control-siRNA or specific p16-siRNA under the control of the U6 promoter in the pRNAT-U6.1 expression vector [20] and also in p16-/- and p16+/+ MEF cells. Figure 1C shows that the down-regulation of p16 decreased the levels of cyclin D1 and E2F1. Indeed, the level of E2F1 was 2 fold lower and the cyclin D1 protein was almost undetectable in p16-silenced cells (Figure 1C). Likewise, in MEF cells, cyclin D1 and E2F1 levels decreased 9 fold and 5 fold in p16-deficient cells as compared to normal cells, respectively (Figure 1C). Moreover, we investigated the expression levels of the *E2F1* and *cyclin D1* mRNAs and we have found that they are also modulated in a p16-dependent manner (Figure 1D). The levels of the *E2F1* and *cyclin D1* mRNAs decreased 3 fold and 2 fold in p16-siRNA-expressing cells as compared to their control counterparts, respectively (Figure 1D, upper panel). These results were confirmed by quantitative real-time RT-PCR (Figure 1D, lower panel). Furthermore, Figure 1D shows that the expression levels of the *E2F1* and *cyclin D1* mRNAs were significantly higher in EH1 and EH2 than in U2OS. In addition, treatment of EH2 cells with IPTG further increased the expression level of the *E2F1* and *cyclin D1* mRNAs (Figure 1D). Together, these results indicate that the expression levels of the cyclin D1 and E2F1 mRNAs and proteins are modulated in a p16-dependent manner in both human and mouse cells.

### p16 controls the turn-over of the cyclin D1 and E2F1 mRNAs

**Figure 2:**
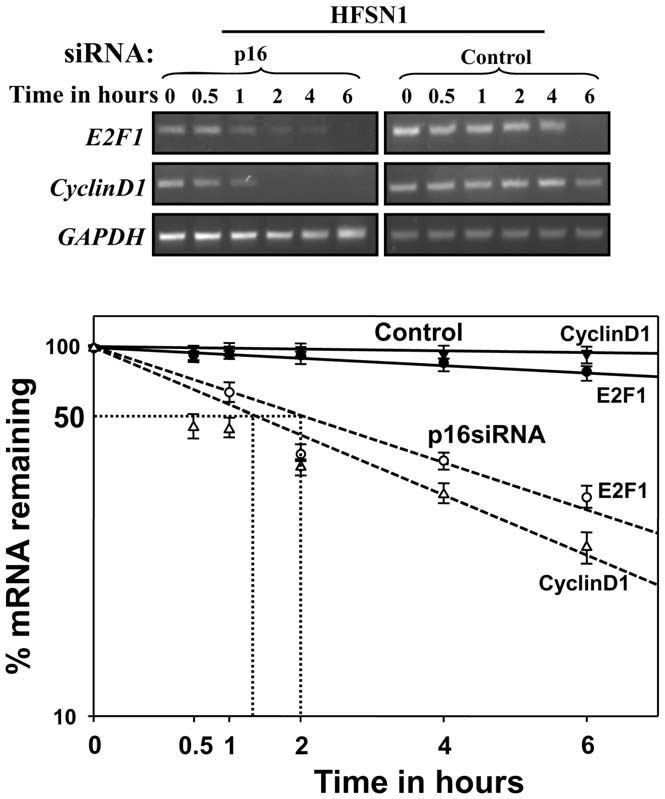
Effect of p16 on the turn-over of the *cyclin D1* and *E2F1* mRNAs. HFSN1 cells were treated with actinomycin D for different periods of time as indicated. Total RNA was extracted and the mRNA levels of *cyclin D1* and *E2F1* were assessed by real-time RT-PCR using specific primers. Continuous lines: HFSN1 expressing control-siRNA, dotted lines: HFSN1 expressing p16-siRNA. Error bars indicate standard errors of 3 different experiments.

Next, we sought to investigate whether p16 has any role in the stability of the *cyclin D1* and *E2F1* mRNAs in the human skin fibroblast HFSN1 cells expressing either p16-siRNA or control-siRNA. Cells were treated with the transcription inhibitor actinomycin D and then reincubated for different periods of time (0–6 hrs). Total RNA was purified and the mRNA levels of *cyclin D1* and *E2F1* were assessed by real time RT-PCR. Figure 2 shows that down-regulation of p16 led to a decrease in *cyclin D1* and *E2F1* half-lives from several hours to an hour and 15 min and 2 hrs, respectively. This result shows that p16 plays a major role in the stability of the *cyclin D1* and *E2F1* mRNAs in normal human skin fibroblast cells.

### p16 negatively controls the mRNA decay-promoting AUF1 protein

**Figure 3:**
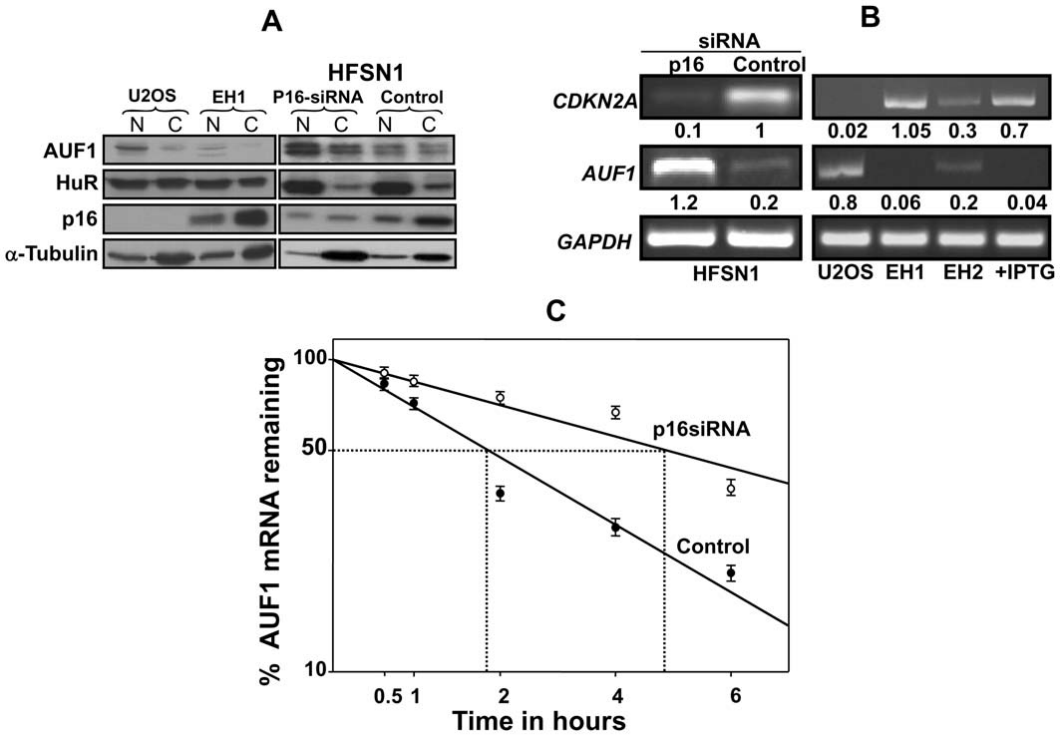
p16 negatively controls the AUF1 expression. (A) Nuclear (N) and cytoplasmic (C) extracts were prepared from the indicated cells and then used for immunoblotting analysis, using the indicated antibodies (B) Total RNA was purified from the indicated cells, and RT-PCR using specific primers for the indicated genes was performed, and the generated fragments were separated on ethidium bromide stained agarose gels. The numbers below the bands indicate the corresponding expression levels relative to GAPDH. These experiments were repeated several times and representative ones are shown. EH2 cells were treated with 1 mM IPTG (C) HFSN1 cells were treated with actinomycin D and then re-incubated for the indicated periods of time. Total RNA was extracted and the amount of mRNA for the indicated genes was assessed using real time RT-PCR. The graph shows the proportion of *AUF1* mRNA remaining post-treatment, and the dotted lines indicate the *AUF1* mRNA half-life. Error bars indicate standard errors of 3 different experiments.

There are RNA binding proteins (RBPs) that bind specific mRNAs and promote either their stabilization or destabilization [29]. HuR and AUF1 are two RBPs that control the decay of several mRNAs, including the *cyclin D1* mRNA [30]. Thereby, we evaluated the effect of p16 on the levels of the HuR and AUF1 proteins in U2OS and EH1 cells as well as in HFSN1 cells expressing either p16-siRNA or control-siRNA. Nuclear and cytoplasmic cell lysates were prepared from these cells and the expression levels of HuR and AUF1 in addition to p16 were assessed. Figure 3A shows that the level of HuR, whose expression is cell cycle regulated [31], [32], was not affected with the status of p16 in both nuclear and cytoplasmic extracts. On the other hand, AUF1, which is essentially nuclear as previously described [30], [33], showed significantly higher levels in the cytoplasmic and the nuclear extracts of p16-defective cells than in their p16-proficient isogenic counterpart cells (Figure 3A). This suggests a role of p16 in the expression of the RNA binding AUF1 protein.

Next, we explored the effect of p16 on the expression of the *AUF1* mRNA. To this end, total RNA was extracted from HFSN1 cells expressing p16-siRNA or control-siRNA as well as from U2OS and its derivatives EH1, EH2 and also from IPTG-treated EH2 cells. RT-PCR utilizing specific primers was used for the assessment of the *AUF1* and *CDKN2A* mRNAs. Figure 3B shows an inverse correlation between the expression levels of the *CDKN2A* and *AUF1* mRNAs. Indeed, the *AUF1* mRNA level is much higher in p16-defective cells (p16-siRNA, U2OS) than in p16-proficient cells (control, EH1, EH2) (Figure 3B). To further elucidate this inverse relationship, EH2 cells were treated with 1 mM of IPTG, which led to 2.5 fold induction in the *CDKN2A* mRNA level. Interestingly, this increase was accompanied by 3 fold decease in the *AUF1* mRNA level (Figure 3B). The increase in the expression level of *AUF1* in p16-siRNA expressing cells was confirmed by quantitative real-time RT-PCR (Figure 1D, lower panel). This indicates that p16 negatively regulates the *AUF1* mRNA.

To investigate whether p16 controls the stability of the *AUF1* mRNA, HFSN1 cells expressing either p16-siRNA or control-siRNA were treated with actinomycin D and then reincubated for different periods of time (0–6 hrs). Total RNA was purified and the mRNA level of *AUF1* was assessed by real time RT-PCR. Figure 3C shows that while the *AUF1* mRNA half-life in the control cells is 1 hr and 50 min, it reached 4 hrs and 45 min in p16-siRNA expressing cells, indicating that p16 plays a major role in the *AUF1* mRNA decay. Together, these results show that p16 negatively controls the expression of *AUF1* by regulating its mRNA turn-over.

### p16 controls the mRNA levels of cyclin D1 and E2F1 through AUF1

**Figure 4:**
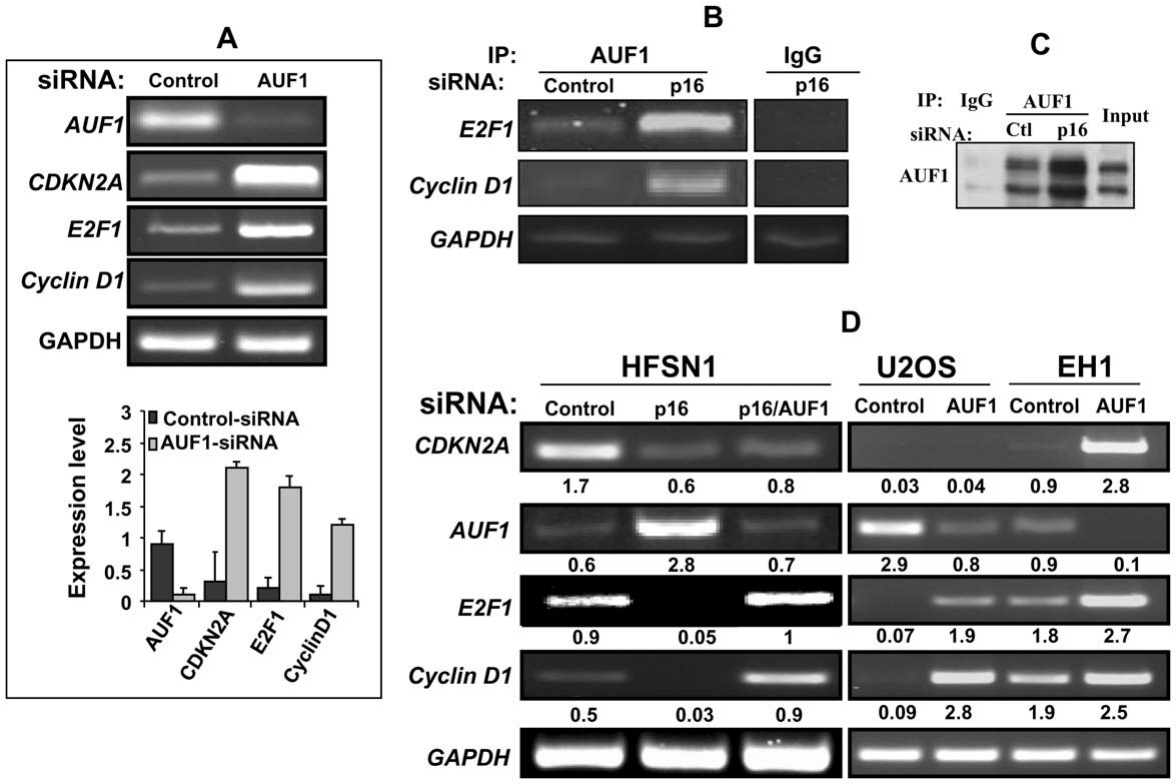
p16 controls the mRNA levels of *cyclin D1* and *E2F1* through AUF1. (A) HFSN1 cells were transfected with plasmids expressing either AUF1-siRNA (pSILENCER-AUF15) or control-siRNA (pSILENCER). 3 days after transfection total RNA was purified from both cells, and RT-PCR (top panel) as well as real time RT-PCR (histogram) using specific primers for the indicated genes were performed. Error bars indicate standard errors of 3 different experiments (B) RNAs bound to the AUF1 protein were isolated by immunoprecipitation from HFSN1 cells expressing either p16-siRNA or control-siRNA using anti-AUF1 antibody or anti-IgG (as control), and then target transcripts were amplified by RT-PCR visualized on Ethidium bromide stained 1% agarose gels. Amplification of the highly abundant GAPDH transcript, which bound IP materials at background levels, was used as loading control. (C) HFSN1 whole cell lysate was immunoprecipitated using the indicated antibodies and then used for immunostaining analysis. (D) Total RNA was purified from p16 proficient (HFSN1 and EH1) and p16-deficient (U2OS and HFSN1 expressing p16-siRNA) cells, expressing either *AUF1*-siRNA or control-siRNA. Transcripts for the indicated genes were detected by RT-PCR, and the corresponding products were visualized on ethidium bromide stained 1% agarose gels. The numbers below the bands indicate the corresponding expression levels. GAPDH was used as internal control. These experiments were repeated several times and representative ones are shown.

Next, we investigated the effect of *AUF1* down-regulation on the mRNA levels of *E2F1* and *cyclin D1* using both RT-PCR and real time RT-PCR. Figure 4A shows that while the level of *AUF1* mRNA was significantly reduced in the *AUF1*-siRNA-expressing cells, the expression level of the *E2F1* and *cyclin D1* mRNAs increased significantly in these cells, as compared to the control cells. These results were confirmed by quantitative real-time RT-PCR (Figure 4A, lower panel). This provides the first indication that the *E2F1* mRNA is also a potential target of AUF1.

Furthermore, we studied the effect of p16 on the binding of AUF1 to the *E2F1* and *cyclin D1* mRNAs using HFSN1 cells expressing either p16-siRNA or control-siRNA. AUF1-mRNAs ribonucleoprotein complexes were obtained by immuoprecipitation (IP) using anti-AUF1 antibody and were used for RT-PCR amplification using specific primers. Figure 4B shows amplification of *cyclin D1* mRNA and also *E2F1* mRNA, indicating the binding of AUF1 protein to *E2F1* mRNA. Importantly, the levels of the *E2F1* and *cyclin D1* mRNAs that were bound to AUF1 were higher in p16-siRNA-expressing cells than in the control cells. This increase correlated with the increase in the immunoprecipitated AUF1 protein in p16-siRNA expressing cells (Figure 4C), confirming what has been shown in figure 3A, and indicating that AUF1 binds specifically to these mRNAs in a p16-dependent manner. This shows that AUF1 plays a major role in mediating the effect of p16 on the expression of *E2F1* and *cyclin D1*.

To further elucidate this role of AUF1, a double knock-down (p16 and *AUF1*) experiment was performed using specific siRNAs in HFSN1 cells. Total RNA was purified and amplified by RT-PCR using specific primers. Figure 4D shows that while the levels of the *cyclin D1* and *E2F1* mRNAs decreased in p16-deficient cells as compared to the control cells, their levels became normal when *AUF1* was down-regulated in p16-defective cells. Similarly, when *AUF1* was knocked-down in the p16-defective U2OS cells, the expression of the two genes increased to a level similar to that observed in p16-proficient EH1 cells (Figure 4D). Together, these data strongly indicate that p16 positively controls the expression of the *cyclin D1* and *E2F1* mRNAs through negative regulation of AUF1.

### The E2F1 3′UTR harbors functional AU-rich elements

**Figure 5:**
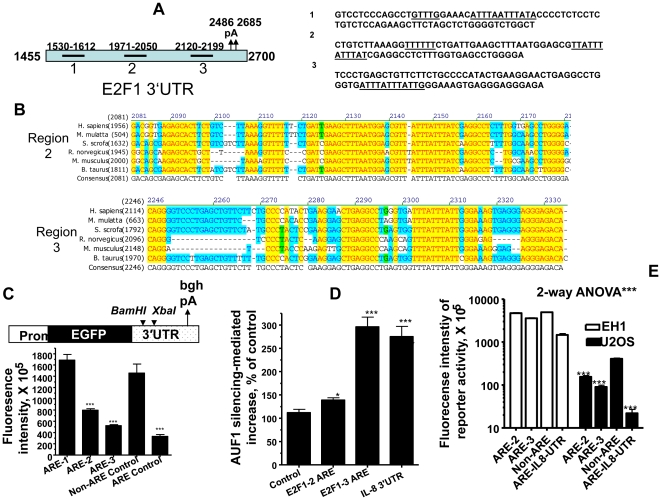
Involvement of ARE in the E2F1 3′UTR and response to AUF1 and p16. (A) Schematic diagram of the E2F1 3′UTR, ARE region sequences, and locations. (B) The E2F1 3′UTR sequences in different species (C) Sequences from the E2F1 3′UTR (*ARE regions 1 to 3*), IL-8 3′UTR (*ARE control*), and a control that lacks ARE were inserted in BamHI/XbaI sites in EGFP expression vector as shown. The Huh7 cell line (2.10^4^ cells per well) in 96-well black clear-bottom microplates were transfected with the different 3′UTR constructs. The reporter activity was assessed after 24 hr using BD bio-imaging apparatus and software. The non-ARE 3′UTR was used as control and its fluorescence activity was taken as 100%. Data are presented as Mean±SEM (n = 4) of % of the control. *** denote *p* values of <0.005 (student t- test) when compared to non-ARE control. (D) Huh7 cells (left panel) or U2OS (right panel) in 96-well microplates were co-transfected with siRNA against AUF1 or scrambled control (50 ng per well) and reporter constructs (25 ng per well) as indicated. Reporter activity was assessed at 48 hr post-transfection. Data (Mean±SEM, n = 4) were presented as % increase in reporter fluorescence due to AUF1 silencing when compared to the fluorescence in control-siRNA-treated cells. * and *** denote *p* <0.05 and <0.005, respectively (student t-test) when compared to non-ARE control. (E) U2OS and EH1 cells (2.10^4^ cells per well) were seeded in 96-well black clear-bottom microplates and then transfected with the different 3′UTR constructs. Reporter activity was assessed as described in (D). ANOVA was performed to compare between U2OS and EH1 data groups.

In order to search for functional mRNA destability element in the E2F1 3′UTR, we analyzed the 3′UTR sequence, which is 1245 nt long, for the presence of conserved and AU-rich element clusters (Figure 5A). Both regions 2 and 3 contain two overlapping AUUUA pentameric repeats (Figure 5A, right panel). These regions were highly conserved among different species (Figure 5B). In order to evaluate the contribution of each of these regions in the *E2F1* mRNA instability, we cloned each of these regions in a vector containing the stable bovine growth hormone (BGH) 3′UTR downstream of the EGFP reporter (Figure 5C, upper panel), and Huh-7 cells were transfected with the three constructs. The reporter activity fused to the 3′UTR with the E2F1 AU-rich regions 2 and 3 was significantly reduced (46% and 64%, respectively) as compared to the control 3′UTR that contains no ARE. On the other hand, the region 1 had no effect (Figure 5C, lower panel). As a positive control, the IL-8 ARE-3′UTR caused 78% reduction of the reporter activity. This EGFP reporter activity system correlated well with ARE- mRNA decay patterns in several cellular models [32], [34], [35]. These results suggest that the *E2F1* 3′UTR bears functional AREs.

### AUF1 controls E2F1 expression in an ARE-dependent manner

Since AUF1 binds the *E2F1* mRNA and activated its decay (Figure 4), we sought to further confirm that AUF1 controls *E2F1* 3′UTR-linked activity in an ARE-dependent manner, using the two *E2F1* ARE-3′UTR constructs that reduced the reporter activity. To this end, we down-regulated the *AUF1* expression using specific siRNA in the Huh cell line. Figure 5D shows that *AUF1* silencing increased the activity of the reporter expressed from the *E2F1* ARE-3′UTR fused constructs. Similarly, as ARE-positive control, IL-8 3′UTR reporter activity increased significantly in response to AUF1 silencing. Together, these results indicate that the AUF1 protein is involved in the post-transcriptional control of *E2F1* through its ARE.

### p16 controls E2F1 expression via ARE-mediated events

To further elucidate the link between p16, AUF1 and E2F1, we assessed the reporter activities described above in both p16-proficient (EH1) and p16-deficient (U2OS) cell lines. Importantly, the reporter activities, for both regions 2 and 3, were more than 12 fold higher in EH1 than in U2OS cells (Figure 5E). This shows the effect of p16 on these activities, and further proofs the role of p16 in the control of *E2F1* mRNA decay.

### Flavopiridol-dependent inhibition of Cdk4/Cdk6 protein kinases does not affect the expression level of AUF1

**Figure 6:**
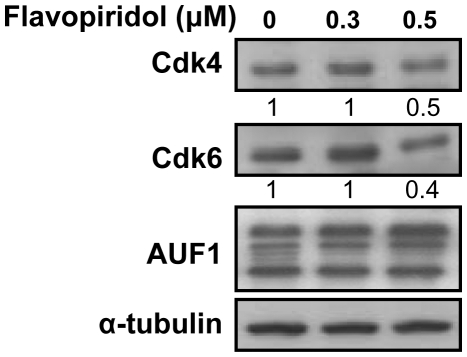
Flavopiridol does not affect the level of the AUF1 protein. HFSN1 cells were treated with 0.3 and 0.5 µM flavopiridol for 24 hrs. Whole cell extracts were prepared and used for western blot analysis using the indicated antibodies.

The two major targets of p16 are the protein kinases Cdk4 and Cdk6. Thereby, we sought to investigate the possible role of these proteins in mediating p16 effect on AUF1. Previous studies have indicated that flavopiridol inhibits the kinase activities of both Cdk4 and Cdk6 [36], [37]. Therefore, HFSN1 cells were treated with flavopiridol (0.3 and 0.5 µM) for 24 hrs. Whole cell extracts were prepared and used to assess the level of Cdk4, Cdk6 and AUF1 by immunoblotting. Figure 6 shows that while the levels of Cdk4 and Cdk6 were halved in response to 0.5 µM flavopiridol, the level of AUF1 remained unchanged. This suggests that Cdk4 and Cdk6 may not be involved in the negative regulation of AUF1 by p16.

### p16 regulates the expression of several genes under the control of E2F1 or AUF1

**Figure 7:**
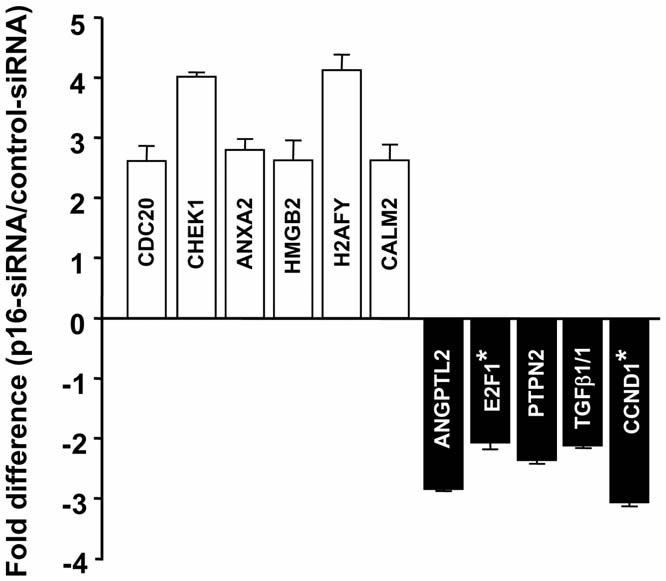
Validation of mRNA levels. The mRNA levels of 11 genes coregulated by both p16 and AUF1 were analyzed by quantitative RT-PCR in HFSN1 cells expressing either p16-siRNA or control-siRNA, and are expressed as fold difference. The error bars represent standard deviation of three different values. *: The fold difference in the expression of these genes was less than 2 in the array data.

The fact that p16 negatively controls the ubiquitous RNA-binding protein AUF1 and the transcription factor E2F1 suggests that p16 might control the expression of several genes. To test this hypothesis, we used oligonucleotide gene expression microarrays to identify genes whose expression is modulated in a p16-dependent manner. Therefore, total RNA was isolated from both p16-siRNA-expressing cells and their control counterparts and was hybridized to microarrays. The difference in gene expression was considered significant only when the variation was ≥2.00. Interestingly, the knocking-down of p16 modulated the expression of 2170 genes, with 1010 (46.5%) were up-regulated and 1160 (53.4%) were down-regulated. Most of these genes (1501) are involved in cell cycle regulation, and the others are implicated in various cancer-related mechanisms such as apoptosis, DNA repair, senescence, angiogenesis and aging. Importantly, the array data showed 4 fold increase in the expression of AUF1 in p16-deficient cells and 46 genes known to be AUF1 targets [38] are also under the control of p16 (Table 1). In addition, 33 genes known to be E2F1 targets [39] are also under the control of p16 (Table 2). To validate the microarray results, the expression level of 11 genes was assessed by quantitative RT-PCR. Figure 7 shows that the expression of all the analyzed genes is modulated in a p16-dependent manner, confirming the microarray data. This shows that the expression of a large number of genes is indeed under the control of the tumor suppressor p16 protein.

**Table 1:** List of genes under the control of both p16 and AUF1.

Affymetrix probesets	Gene Fold Change	Gene Symbol	Gene Name	Biological Function
208051_s_at	3.29873	PAIP1	Polyadenylate binding protein-interacting protein 1	Regulation of mRNA and protein stability and metabolism
201019_s_at	2.02846	EIF1A	Eukaryotic translation initiation factor 1A	Regulation of cellular protein metabolic process and translation
203316_s_at	−3.2073	RPE	Ribose-5-phosphate-3-epimerase	RNA splicing
202017_at	−2.00067	EPHX1	Epoxide hydrolase 1, microsomal (xenobiotic)	Response to toxin
208808_s_at	2.47779	HMGB2	High-mobility group box 2	DNA packaging, gonad development, negative regulation of macromolecule metabolic process
205394_at	2.08141	CHEK1	CHEK1 checkpoint homolog (S.pombe)	G2/M transition of mitotic cell cycle, protein phosphorylation, DNA damage response
202870_s_at	4.46627	CDC20	CDC20 cell division cycle 20 homolog (S.cerevisiae)	Proteasomal ubiquitin-dependent protein catabolic process
228940_at	−2.1415	NDUFB5	NADH dehydrogenase (ubiquinone) 1 beta subcomplex 5	Response to oxidative stress
211713_x_at	2.71946	KIAA0101	KIAA0101 gene product	Skeletal system development
201344_at	2.30847	UBE2V2	Ubiquitin-conjugating enzyme E2 variant 2	Chromatin organization, ubiquitin-dependent protein catabolic process
213661_at	−3.30605	DKFZP547E2110	Anti-silencing function 1A	Cell cycle regulation
204905_s_at	2.1388	EEF1A1	Eukaryotic translation elongation factor 1alpha 1	Positive regulation of response to stimulus
201437_s_at	2.71295	EIF4A2	Eukaryotic translation initiation factor 4A, isoform 2	Posttranscriptional regulation of gene expression
213004_at	−3.30579	ANGPTL2	Angiopoietin-like 2	Negative regulation of programmed cell death
1555882_at	−2.45564	SPINL	Spinster-like protein	Spindle organization
40524_at	−2.20011	PTPN2	Protein tyrosine phosphatase, non-receptor type 2	Enzyme linked receptor protein signaling pathway
1559263_s_at	2.2216	PPIL4	Peptidylprolyl isomerase (cyclophilin)-like 4	RNA processing
214500_at	4.17384	H2AFY	H2A histone family, member Y	Cellular response to stress and DNA damage
204023_at	2.53988	RFC4	Replication factor c (activato 1) 4, 37kDa	Nucleotide-excision repair, response to DNA damage
202094_at	4.22242	BIRC5	Baculoviral IAP repeat-containing 5 (survivin)	G2/M transition of mitotic cell cycle, Apoptosis regulation
1555495_a_at	2.0944	SDCCAG16	Serologically defined colon cancer antigen 16	Response to toxin
211985_s_at	2.46326	CALM2	Calmodulin 2 (phosphorylase kinase, delta)	Response to calcium ion, regulation of cell division
202274_at	−2.14338	ACTG1	Actin, gamma 1	Vascular process in circulatory system
208969_at	2.13464	NDUFA5	NADH dehydrogenase (ubiquinone) 1 alpha subcomplex 5	Response to oxidative stress
201433_s_at	2.11347	PTDSS1	Phosphatidylserine synthase 1	Regulation of cell proliferation
236561_at	−2.11136	TGFB1/1	Transforming growth factor beta 1 induced transcript 1	Respiratory system development
203396_at	2.04501	PSMA1	Proteasome subunit, alpha type 1	Regulation of protein modification and metabolism
209988_s_at	−2.14241	ASCL1	Achaete-scute complex-like 1 (Drosophila)	Cell proliferation, positive regulation of transcription
218350_s_at	2.54972	GMNN	Geminin, DNA replication inhibitor	Regulation of DNA replication and cell cycle
203180_at	−2.38611	ALDH3A1	Aldehyde dehdrogenase 3 family, member A1	Regulation of apoptosis
1554161_at	2.71614	SLC25A17	Solute carrier family 25 member 17	Response to inorganic substance, nucleotide biosynthesis
214060_at	−3.90345	SSBP1	Single-stranded DNA binding protein	DNA replication, cellular component morphogenesis
242210_at	−2.99625	ZNF24	Zinc finger protein 24 (KOX 17)	Regulation of transcription
201120_s_at	−2.33754	PGRMC2	Progesterone receptor membrane component 2	Regulation of cell adhesion
1553313_s_at	−3.15507	SLC5A6	Solute carrier family 5 member 6	Response to calcium ion
203152_at	2.02172	MRPL4	Mitochondrial ribosomal protein L4	RNA processing, translation
2028_s_at	−1.26292	**E2F1** *	E2F1 transcription factor 1	Regulation of cell cycle, regulation of transcription
208712_at	−1.68361	**CCND1** *	CyclinD1	Cell cycle checkpoint
213182_x_at	−3.10006	CDKN1C	P57.KIP2	Regulation of kinase activity
227299_at	2.37098	CCNI	Cyclin I	Regulation of cell cycle process
210609_s_at	2.3745	TP53I3	Tumour protein p53 inducible protein 3	Positive regulation of programmed cell death
225912_at	−2.22501	TP53INP1	Tumour protein p53 inducible nuclear protein 1	Induction of apoptosis
210734_x_at	−2.47494	MAX	MYC associated factor x	RNA biosynthetic process
1567457_at	2.40059	RAC1	Ras-related C3 botulinum toxin substrate 1	Regulation of phosphoinositide 3-kinase activity
201437_s_at	2.12563	EIF4E	Eukaryotic translation initiation factor 4E)	Posttranscriptional regulation of gene expression
1568126_at	2.09032	ANXA2	Annexin A2	Angiogenesis, extracellular matrix organization

*: The variation in the expression of these genes was less than 2 fold in microarray analysis data. However, the variation was significant when RT-PCR was used for assessment, as shown in figure 1D and figure 7.

**Table 2:** List of genes under the control of both p16 and E2F1.

Affymetrix probesets	Gene Fold Change	Gene Symbol	Gene Name	Biological Function
211464_x_at	−2.108381	Casp6	Caspase 6, apoptosis-related cysteine peptidase	Regulation of protein localization, regulation of apoptosis
205681_at	6.50843	Bcl-2	B-cell CLL/lymphoma 2	Negative regulation of apoptosis
1861_at	−2.12347	BAD	BCL2-associated agonist of cell death	Positive regulation of apoptosis
207535_s_at	3.67377	NFκB	Nuclear factor of kappa light polypeptide gene enhancer in B-cells (p64/p100)	Adaptive immune response, extracellular matrix organization
211828_s_at	−2.29209	TRAF2	TNF receptor-associated factor 2	RNA splicing
208712_at	−1.68361	CCND1	CyclinD1	Cell cycle checkpoint
205034_at	7.27143	CCNE2	CyclinE	Regulation of cyclin-dependent protein kinase activity, cell cycle checkpoint
213226_at	4.05707	CCNA	CyclinA	G2/M transition DNA damage checkpoint
202534_x_at	5.08339	DHFR	Dihydrofolate reductase	Nucleotide biosynthetic process
214056_at	2.07083	Mcl1	Myeloid cell leukemia sequence 1 (BCL2-related)	Response to cytokine stimulus, cell fate determination, negative regulation of cell death
200893_at	2.26377	SFRS10	Splicing factor, arginine/serine-rich 10 (transformer 2 homolog, Drosophila)	RNA processing
201129_at	2.59299	SFRS7 (SR protien 9G8)	Splicing factor, arginine/serine-rich 7, 35kDa	RNA processing
201586_s_at	3.57618	SFPQ (PSF)	Splicing factor proline/glutamine-rich (polypyrimidine tract binding protein )	DNA recombination
1552627_a_at	−2.37653	ARHGAP4	Rho GTPase activating protein 4	Positive regulation of mesenchymal cell proliferation, regulation of cell migration
205024_s_at	3.03581	RAD52	RAD52 homolog (RecA homolog, E. coli) (S. cerevisiae)	Sister chromatid cohesion, cellular response to stress
204127_at	4.59846	RFC3	Replication factor C (activator 1) 3, 38kDa	DNA strand elongation during DNA replication, nucleotide-excision repair, response to toxin
223746_at	−2.01097	STK15	Serine/threonine kinase 15	Protein amino acid autophosphorylation, regulation of apoptosis
221085_at	7.83536	TNFSF9	Tumor necrosis factor (ligand) superfamily, member 9	Tumor necrosis factor receptor binding, cytokine activity
213575_at	−2.77451	TRA1	Transformer-1	mRNA binding and splicing
210563_x_at	−2.18188	CFLAR (FLIP)	CASP8 and FADD-like apoptosis regulator	Cysteine-type peptidase activity
205192_at	−2.13176	MAP3K14	Mitogen-activated protein kinase kinase kinase 14	Cell cycle regulation, protein amino acid phosphorylation
221695_s_at	−2.9927	MAP3K5	Mitogen-activated protein kinase kinase kinase 5	Cell cycle regulation, protein amino acid phosphorylation
208378_x_at	−2.17358	FGF-2	Fibroblast growth factor 2	Regulation of cell division
210973_s_at	−2.04823	FGFR3	Fibroblast growth factor receptor 2	Positive regulation of cell proliferation
217279_x_at	−2.00664	MMP16	Matrix metallopeptidase 16 (membrane-inserted)	Regulation of cell adhesion
211527_x_at	−4.52681	VEGF-B	Vascular endothelial growth factor B	Regulation of cell division, transmembrane receptor protein tyrosine kinase signaling pathway, positive regulation of gene expression
201005_at	−2.97704	CD9	CD9 antigen (p24)	Wound healing, cellular component morphogenesis, negative regulation of cell proliferation
217294_s_at	−7.39815	ENO2	Enolase 2	Hydro-lyase activity, protein and nucleic acid binding, transcription cofactor activity, metabolism
225669_at	2.08248	IFNA2	Interferon (alpha, beta and omega) 2	Posttranscriptional regulation of gene expression, cell cycle arrest, cell death
228325_at	−3.80088	KIAA0455	KIAA0455	Skeletal system development
224762_at	2.14831	KIAA0767	KIAA0767	Sskeletal system development
202283_at	−2.59665	SERPINF2	Serpin peptidase inhibitor, clade F (alpha-2 antiplasmin, pigment epithelium	Regulation of inflammatory response
208905_at	2.32975	CYCS	Cytochrome c, somatic	Cellular macromolecule catabolic process

### p16-dependent modulation of E2F1 affects doxorubicin-induced apoptosis and apoptotic proteins

**Figure 8:**
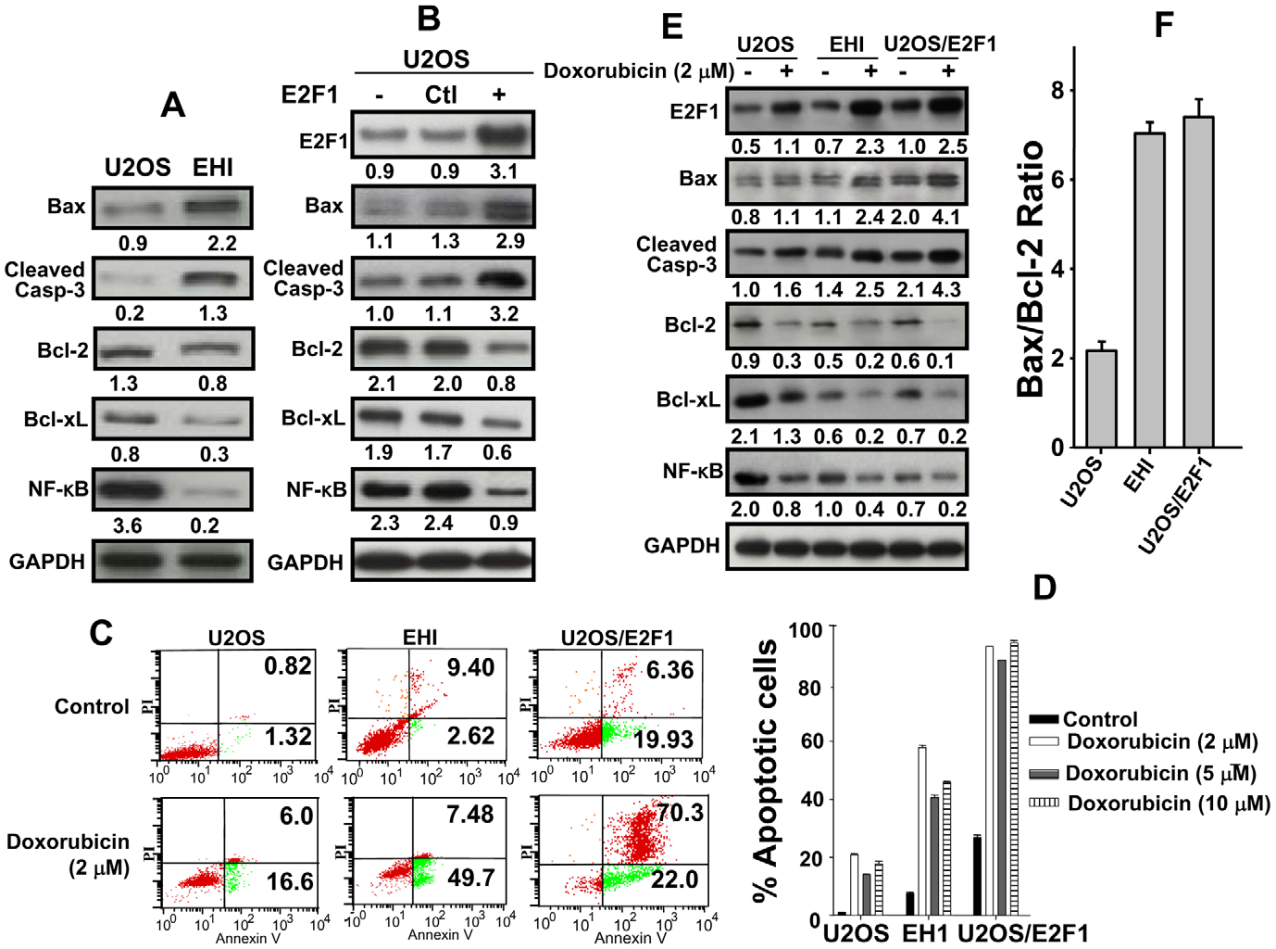
p16 modulates apoptosis through E2F1. (A) Western blots showing the expression of the indicated pro-and anti-apoptotic proteins in U2OS and EHI. (B) Western blots showing the expression of the indicated proteins in U2OS, U2OS cells stably transfected with plasmids encoding either scrambled sequence (ctl) or E2F1 (+). The numbers below the bands indicate the corresponding expression levels. (C) U2OS, EH1 and E2F1-expressing U2OS cells were either mock-treated or challenged with doxorubicin (2 µM) and then re-incubated for 72 hrs. Cells were then divided into two groups; one was used to analyze cell death by annexinV/PI flow cytometry. The numbers in the charts indicate the proportions of early and late apoptosis. (D) Histogram showing the proportions of apoptosis (early+late) induced by different doses of doxorubicin. The error bars represent standard deviation of three different experiments. (E) The second group of cells was used to assess the level of the indicated proteins by immunoblotting. (F) Graph showing the Bax/Bcl-2 ratio in the indicated cells after treatment with doxorubicin. Error bars represent standard deviation of at least three different experiments.

Next, we studied the effect of p16-dependent modulation of E2F1 level on the expression of apoptotic-regulatory proteins, known to be under the control of E2F1 [40]. Figure 8A shows that the levels of the pro-apoptotic proteins Bax and cleaved Caspase-3 are respectively 2.4 and 6.5 fold higher in EH1 as compared to U2OS. On the other hand, the expression levels of the anti-apoptotic proteins Bcl-_2_, Bcl-_xL_ and NF-κB were respectively 1.6, 2.6 and 18 fold lower in EH1 as compared to U2OS. This indicates that the expression level of various apoptosis proteins is modulated in a p16-dependent manner. To show that these variations are E2F1-dependent, E2F1 was ectopically introduced into the p16-defective U2OS cells and whole cell extracts were prepared from U2OS and U2OS expressing either the control plasmid or E2F1-expressing plasmid. Figure 8B shows that the expression level of E2F1 increased 3.4 fold in E2F1-expressing U2OS cells as compared to the control. Consequently, there was a clear correlation between the E2F1 expression level and the expression of the pro- and anti-apoptotic proteins. In E2F1-expressing U2OS cells, the expression levels of Bax and cleaved Caspase-3 were 2.2 and 2.9 fold higher than in the control cells, while the expression levels of Bcl-2, Bcl-xL and NF-κB were 2.5, 2.8 and 2.6 fold lower than in the control, respectively (Figure 8B). This suggests that p16-dependent modulation of the expression of the apoptotic genes could be mediated through E2F1.

To further investigate this, U2OS cells expressing control plasmid or E2F1-expressing plasmid as well as the p16-proficient isogenic EH1 cells were either sham-treated or challenged with different doses of doxorubicin for 3 days, and the level of apoptosis was assessed by Annexin V/flow cytometry. Figure 8C and Figure 8D show that the proportion of spontaneous apoptosis in U2OS cells (2.14%) was enhanced by the over-expression of E2F1 (26.29%). Similarly, doxorubicin-induced apoptosis was higher in U2OS cells expressing E2F1 than in the control cells (Figure 8C and Figure 8D). Indeed, while apoptosis in response to doxorubicin was only 20% in U2OS cells, it reached about 45% in EH1 cells and 66% in U2OS cells wherein E2F1 was over-expressed (Figure 8D). This indicates that the expression of E2F1 in p16-defective cells restores p16-related apoptosis, which suggests that the apoptotic effect of p16 is mediated through the E2F1 transcription factor. To confirm this, we investigated the effect of p16 and doxorubicin on the level of pro- and anti-apoptotic proteins in U2OS, EH1 and U2OS expressing E2F1. Figure 8E shows that doxorubicin treatment (2 µM for 3 days) up-regulated the E2F1 protein in the 3 different cell lines. However, the level of E2F1 is more than 2 fold higher in EH1 and U2OS expressing E2F1 than in U2OS. Importantly, doxorubicin effect on the cleaved caspase-3 and Bax levels was only marginal in U2OS, but it led to 2 fold increase in the p16-proficient EH1 cells and U2OS cells expressing E2F1, as compared to their respective non-treated cells (Figure 8E). Concomitantly, the levels of NF-κB, Bcl-2 and Bcl-xL were reduced in EH1 and U2OS-expressing E2F1 cells as compared to their levels in U2OS, and doxorubicin treatment decreased their levels in the 3 cell lines, but its effect was more pronounced in p16-proficient and E2F1-expressing U2OS cells (Figure 8E). Furthermore, the level of the Bax/Bcl-2 ratio significantly increased in p16-proficient EH1 cells and also in p16-deficient cells expressing E2F1 as compared to p16-deficient U2OS cells (Figure 8F). This further shows the role of p16 in inducing doxorubicin-dependent apoptosis, and that E2F1 overexpression overcomes p16-deficiency in this process.

## Discussion

**Figure 9:**
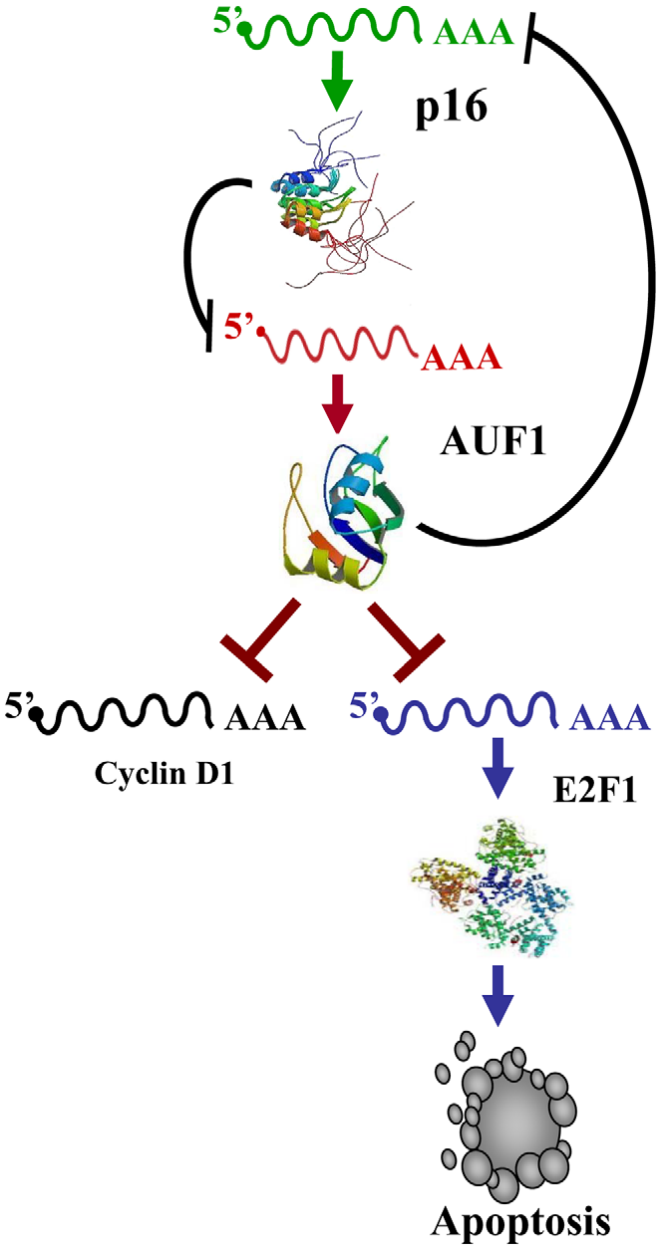
Schematic representation of the p16-related regulation of AUF1, E2F1 and CyclinD1. See text for details.

In the present report we have shown that p16 is not only a cell cycle checkpoint protein, but it is also a master regulator of gene expression in absence of cellular stresses. Indeed, p16 negatively regulates the ubiquitous RNA-binding protein AUF1 through controlling the turn-over of its mRNA. Furthermore, p16 positively regulates the expression of cyclin D1 and the transcription factor E2F1 at the post-transcriptional level. We have also shown the presence of 3 AU-rich element clusters in the *E2F1* mRNA (Figure 5), which provides the first indication that the oncogene/tumor suppressor gene *E2F1* codes for an ARE-mRNA that is under the control of AUF1. It is noteworthy that the status of p16 had no effect on the level of the cell cycle regulated HuR protein (Figure 3A) and also on the cell proliferation protein PCNA (Figure 1A), which ruled out the possible implication of the cell cycle, and indicates that the modulation in the expression of *cyclin D1* and *E2F1* is rather p16-dependent. The fact that AUF1 negatively controls p16 [41], points to the presence of a negative feedback loop between the tumor suppressor p16 and the RNA binding AUF1 protein (Figure 9). While AUF1 is known to regulate p16 through binding and promoting the destabilization of its mRNA [41], [42], it still unclear how p16 regulates the expression of AUF1. We have shown here that p16 controls the stability of the AUF1 mRNA. However, p16 is not known to be an RNA binding protein. Therefore, we hypothesized that p16 could affect the AUF1 mRNA decay through one of its major targets Cdk4 and Cdk6 [7]. However, the inhibition of both kinases by Flavopiridol did not affect the level of the AUF1 protein (Figure 6), suggesting that p16-dependent regulation of AUF1 may not be mediated through Cdk4 or Cdk6. Consequently, it is possible that p16 regulates AUF1 expression through another RNA-binding protein or through specific miRNA(s). We are currently investigating these two possibilities to elucidate the exact mechanism(s) whereby p16 controls *AUF1* mRNA turn-over.

Concerning the link between p16 and cyclin D1, Wang et al. have previously shown that down-regulation of AUF1 by specific siRNA up-regulated p16 protein and concomitantly increased the level of cyclin D1 [41]. Furthermore, it has been shown that in senescent cells there is concomitant increase in the levels of both p16 and cyclin D1, while cyclin A and cyclin B1 levels decrease [41], [43]. These data are in keeping with the results presented here, showing that cyclin D1 expression is modulated in a p16-dependent manner at least in human skin fibroblasts. In cancer cells, in addition to the osteosarcoma U2OS cells, DOAY and other p16-defective medulloblastoma cells also showed undetectable or very low levels of cyclin D1 (data not shown), which further supports this link between p16 and cyclin D1.

The fact that p16 controls the expression of both AUF1 and E2F1, two important transcription regulators, suggested that this CDKI can also modulate the expression of a plethora of genes. Using microarray analysis we have shown that p16 controls the expression of more than 2000 genes involved in various cellular metabolisms, and most of them are implicated in cell proliferation and carcinogenesis. Importantly, 46 of these genes are members of the AUF1 regulon, and 33 genes are co-regulated by p16 and E2F1 (Table 1 and Table 2). Among these genes there is another master transcription regulator, NF-κB. The mRNA and protein levels of this gene increased 3.6 fold and 18 fold in p16-deficient cells as compared to their respective controls, respectively (Figure 8A and Table 2). This effect seems to be E2F1-dependent since the overexpression of E2F1 decreased the level of NF-κB in p16-deficient U2OS cells (Figure 8B). Together, these results suggest that p16 exerts a negative control on the expression of NF-κB, through the transcription factor E2F1. This relationship between p16 and NF-κB is of great importance since NF-κB regulates the expression of a large number of genes important for apoptosis, tumorigenesis, viral replication, inflammation and various immune responses [44], which could suggest a key role of p16 in all these processes.

Annexin A2 (ANXA2) is another important cancer gene under the control of p16 and AUF1 (Table 1). The level of the ANXA2 gene increased 2 fold in p16-deficient cells, and it is known that its expression is elevated in a wide range of cancers [45], including melanomas wherein p16 is usually inactive [14], [46]. This suggests an important role of p16 in the increase in the ANXA2 expression level in cancer cells.

What are the consequences of p16-dependent modulation of E2F1 expression level on cell proliferation and/or cell dearth? In fact, the modulation in p16/E2F1 expression did not affect cell proliferation nor the expression of PCNA (Figure 1A), which is under direct control of E2F1 [40]. The role of E2F1 in apoptosis is attributed mainly to E2F1-dependent up-regulation of various pro-apoptotic genes, like caspases and Bax and/or inhibiting survival signals, in particular those mediated by the transcription factor NF-κB or Bcl-2 [47]. Importantly, we have found that p16 is also a positive regulator of the pro-apoptotic protein Bax and an inhibitor of the anti-apoptotic proteins Bcl-2, Bcl-xL and NF-κB. This effect is E2F1-mediated, since the over-expression of this protein restored the normal expression of these apoptosis-related proteins in p16-defective cells. This suggests that p16 is also a modulator of apoptosis through the E2F1 protein (Figure 9). To further elucidate this, we have shown that the over-expression of the E2F1 protein enhances spontaneous as well as doxorubicin-induced apoptosis in the osteosarcoma p16-defective U2OS cells. Similar results were obtained by the ectopic expression of p16 in the U2OS cells (EH1), indicating that p16 sensitizes the osteosarcoma cells to doxorubicin through E2F1. Furthermore, we have previously shown that p16 modulates apoptosis in response to both UV light and cisplatin [23]. Together, these results show that, like E2F1, p16 plays also a major role not only in cell growth but also in cell death [5].

In summary, these results provide the first clear indication that the cyclin-dependent kinase inhibitor p16 protein also modulates the expression of several key transcription regulators such as AUF1, E2F1 and NF-κB, and other important genes directly or indirectly involved in cancer development and spread. This sheds more light on the molecular basis of the tumor suppressor function of this protein.
